# Dynamic immune cell profiling identified natural killer cell shift as the key event in early allograft dysfunction after liver transplantation

**DOI:** 10.1111/cpr.13568

**Published:** 2023-10-31

**Authors:** Di Lu, Xinyu Yang, Linhui Pan, Zhengxing Lian, Winyen Tan, Jianyong Zhuo, Modan Yang, Zuyuan Lin, Qiang Wei, Jun Chen, Shusen Zheng, Xiao Xu

**Affiliations:** ^1^ Zhejiang University School of Medicine Hangzhou China; ^2^ Key Laboratory of Integrated Oncology and Intelligent Medicine of Zhejiang Province Hangzhou China; ^3^ Institute of Organ Transplantation Zhejiang University Hangzhou China; ^4^ Department of Hepatobiliary and Pancreatic Surgery Shulan (Hangzhou) Hospital Hangzhou China

## Abstract

Early allograft dysfunction (EAD) is a life‐threatening and fast‐developing complication after liver transplantation. The underlying mechanism needs to be better understood, and there has yet to be an efficient therapeutic target. This study retrospectively reviewed 109 patients undergoing liver transplantation, with dynamic profiling of CD3/4/8/16/19/45/56 on the peripheral immune cells (before transplant and 2–4 days after). Altogether, 35 out of the 109 patients developed EAD after liver transplantation. We observed a significant decrease in the natural killer cell proportion (NK cell shift, *p* = 0.008). The NK cell shift was linearly correlated with cold ischemic time (*p* = 0.016) and was potentially related to the recipients' outcomes. In mouse models, ischemic/reperfusion (I/R) treatments induced the recruitment of NK cells from peripheral blood into liver tissues. NK cell depletion blocked a series of immune cascades (including CD8+ CD127+ T cells) and inhibited hepatocyte injury effectively in I/R and liver transplantation models. We further found that I/R treatment increased hepatic expression of the ligands for natural killer group 2 member D (NKG2D), a primary activating cell surface receptor in NK cells. Blockade of NKG2D showed a similar protective effect against I/R injury, indicating its role in NK cell activation and the subsequent immunological injury. Our findings built a bridge for the translation from innate immune response to EAD at the bedside. Peripheral NK cell shift is associated with the incidence of EAD after liver transplantation. NKG2D‐mediated NK cell activation is a potential therapeutic target.

## Graphical abstract

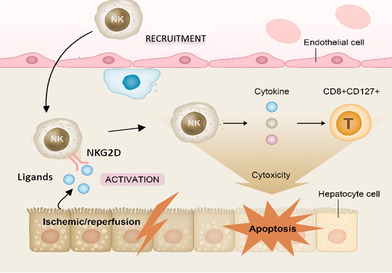

## 1 INTRODUCTION

Liver transplantation is currently the most effective treatment for end‐stage liver disease.^1^, ^2^ With the remarkable development in techniques and strategies of liver transplantation, the incidence of post‐operative complications has been reduced profoundly, along with the significant improvement in the long‐term survival of patients throughout the years. However, owing to the rapid‐growing liver transplant candidates, the use of marginal donor liver has increased dramatically during the past decade.^3^ This has led to reduced graft quality and increased incidence of severe complications after transplantation, including early allograft dysfunction (EAD). EAD describes liver grafts with poor initial function after liver transplantation. Patients with EAD are associated with inferior survival as EAD may develop into primary graft non‐function, resulting in patient death or re‐transplantation.^4^, ^5^, ^6^ Due to the lack of specific animal models, the underlying mechanism for EAD was poorly understood. To avoid donor organ wastage resulting from EAD, finding effective biomarkers and therapeutic targets for this fast‐developing but life‐threatening complication is of great clinical value.

During organ procurement and transplantation, the liver undergoes a series of traumatic events, including cold storage, warm ischemia, implantation and reperfusion injury, all of which adversely impact the graft and may increase the risk of EAD. Upon liver reperfusion, the graft is connected to the systemic circulation system, and the immunological interaction between the recipient and donor liver occurs automatically.^7^, ^8^ The liver injury may affect both the hepatic and systemic immunology after that. To date, no report has evaluated EAD‐related changes in immune cell composition. Therefore, our study aimed to profile the dynamic changes of peripheral immune cells to identify a unique immunological event in patients with EAD after transplantation.

## 2 RESULTS

### 2.1 Peripheral natural killer (NK) cell shift is associated with EAD

**FIGURE 1:**
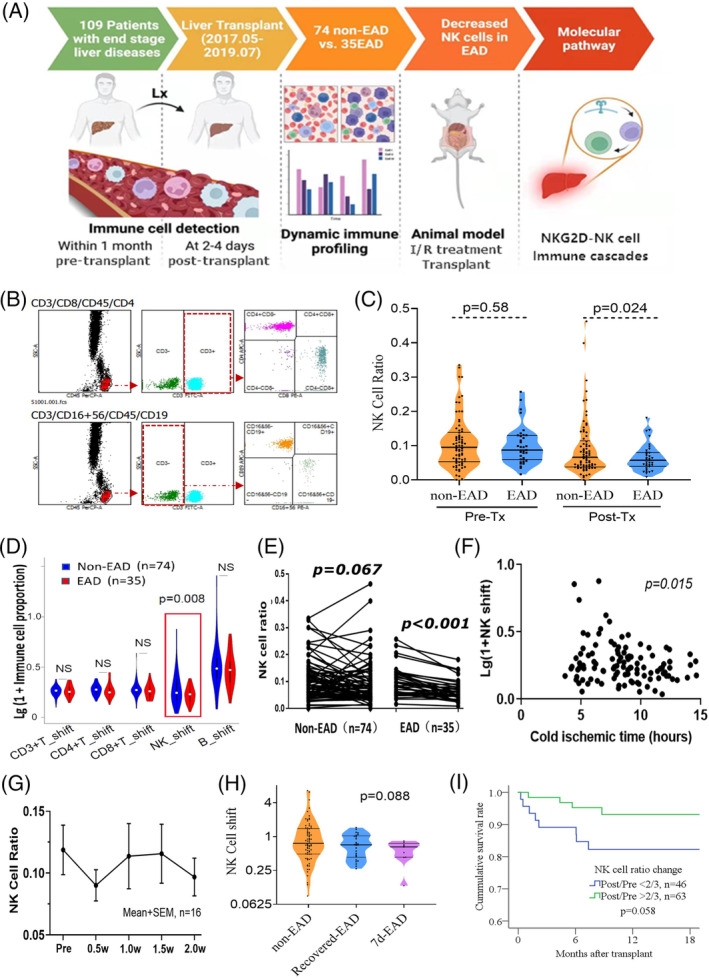
Study workflow and peripheral immune cell profiling. (A) Study workflow: Among the 109 patients undergoing liver transplantation enrolled, testing immune profiling within 1 month before transplantation and at 2–4 days after transplantation, 74 cases did not have EAD (non‐EAD group) and 35 cases had EAD (EAD group). Animal models of ischemia reperfusion injury (IRI) and rat liver transplantation were used for further validation. (B) Gating in flow cytometry was performed automatically. We firstly gated CD45 to identify the CD45+ lymphocytes. The CD45+ lymphocytes were then gated by CD3. When analysing CD19 and CD56, only those CD3− lymphocytes were enrolled. Meanwhile, only those CD3+ lymphocytes were enrolled in the analysis of CD4 and CD8. The results will be verified and fine‐tuned by a professional technician. (C) Dynamic comparison of natural killer cell proportions between the non‐EAD and EAD groups. (D) The vioplot showed the difference in immune profiling dynamic shift between the non‐EAD and EAD groups. The dynamic shift was obtained by calculating the ratio of post‐operative and preoperative proportions. (E) The NK cell proportion shift in the non‐EAD group and EAD group. (F) The correlation between NK cell shift and cold ischemia time. (G) The dynamic curve of NK cell proportion from pre‐transplant to 2 weeks post‐transplant. (H) NK cell shift in different groups. Non‐EAD group refers to patients who did not develop EAD. The recovered‐EAD group refers to patients who had EAD but recovered within 7 days after transplantation. The 7d‐EAD group refers to those who did not recover within 7 days after transplantation. (I) Kaplan–Meier estimates of cumulative survival rate. NK cell shift <2/3 was associated with relatively poorer survival after transplantation. EAD, early allograft dysfunction.

This study enrolled 109 patients undergoing donation after circulatory death (DCD) liver transplantation. The general workflow is shown in Figure 1A. To monitor the acute immunological responses, we collected immune cell profiling (CD3/4/8/16/19/45/56) data before transplantation (within 30 days) and 2–4 days after, respectively. We also collected clinical information, including donor and recipient demographics, blood type, body mass index (BMI), the steatotic status of the donor liver, graft weight and graft weight ratio, pre‐transplant recipient model for end‐stage liver diseases score and neutrophil to lymphocyte ratio, platelet to lymphocyte ratio, operation time, cold ischemic time (CIT), warm ischemic time, blood loss, post‐operative alanine aminotransferase (ALT), aspartate aminotransferase (AST), international normalised ratio (INR) within 7 days, perioperative red blood cell, white blood cell, platelet, monocyte, lymphocyte and neutrophil counts. There were 80 male and 29 female patients. Their age ranged from 23 to 75 years old, averaging 49.3. The average follow‐up time was 12.5 months.

EAD was defined as the presence of one or more of the following: bilirubin ≥10 mg/dL on day 7, INR ≥1.6 on day 7, and AST or ALT >2000 IU/L within the first 7 days.^9^ Altogether, 35 out of the 109 patients developed EAD after liver transplantation. The relatively high ratio of EAD is considered as a result of the usage of DCD donor livers, which adversely impacts organ function after transplantation.^10^

Table 1 shows the clinical characteristics associated with EAD: donor BMI, donor graft weight, fatty liver change and CIT (*p* < 0.05). Tables 2 and 3 show the absolute number and proportion of immune cell components before the transplant and 2–4 days after. The gating of CD3/4/8/16/19/45/56 in flow cytometry is shown in Figure 1B. We compared the profiles of the patients with EAD (*n* = 35) and those without (*n* = 74). In the pre‐transplant data, the neutrophil‐to‐white blood cell ratio (NWR) was significantly higher (*p* = 0.048). In contrast, the lymphocyte‐to‐white blood cell ratio was considerably lower in the EAD patients (*p* = 0.045). In the post‐transplant data, the proportion of NK cells (CD3− CD16/CD56+ lymphocytes to CD45+ lymphocyte percentage) was lower in the EAD patients (*p* = 0.024, Figure 1C). We further analysed the dynamic changes of immune cell components (Figure 1D). We found a more significant reduction of NK cell proportion in the EAD patients than those without (*p* = 0.008, Figure 1E). We named it NK cell shift (post‐transplant NK cell proportion to pre‐transplant NK cell proportion).

**TABLE 1:** Clinical characteristics in relation to EAD.

	Non‐EAD (*n* = 74)	EAD (*n* = 35)	*p* *
Donor characteristics			
Sex (male)	77.0%	91.4%	0.11
Age (years)	45.0 ± 13.5	45.5 ± 12.3	0.86
BMI (kg/m^2^)	22.9 ± 3.0	24.4 ± 3.2	0.018
Graft weight (g)	1139.1 ± 320.7	1151.3 ± 403.4	0.018
Fatty liver change (>5%)	10.8%	37.1%	0.003
WIT (min)	8.1 ± 7.5	7.9 ± 7.0	0.89
CIT (h)	8.1 ± 2.5	9.3 ± 3.0	0.026
GWR (%)	21.7 ± 6.2	22.9 ± 6.7	0.35
ABO incompatibility	13.5%	17.1%	0.77
Recipient characteristics			
Liver failure	17.6%	22.9%	0.51
Sex (male)	62.2%	68.6%	0.49
Age (years)	49.8 ± 10.3	48.3 ± 11.1	0.47
BMI (kg/m2)	22.9 ± 3.3	24.3 ± 3.8	0.055
MELD score	21.3 ± 9.9	20.4 ± 10.5	0.98
NLR	6.8 ± 8.6	8.8 ± 8.7	0.26
PLR	97.2 ± 71.1	102.9 ± 67.9	0.70
Operative information			
Operation time (h)	5.4 ± 1.0	5.7 ± 1.4	0.18
Blood loss (L)	1.4 ± 1.6	1.3 ± 0.8	0.68
Transfused RBC (u)	5.3 ± 5.4	5.0 ± 3.9	0.76
Intraoperative autotransfusion (ml)	329.7 ± 650.8	175.7 ± 388.9	0.13
Anhepatic time (min)	60.0 ± 14.8	62.0 ± 25.3	0.47
Interval between portal and arterial reperfusion	22.5 ± 26.7	27.5 ± 25.6	0.25
Post‐operative complication			
Bleeding	5.4%	5.7%	1.00
Arterial thrombosis	1.4%	0	1.00
Biliary complications	13.5%	8.6%	0.54
Infection	17.6%	28.6%	0.21
Primary nonfunction	0	2.8%	0.32

Abbreviations: BMI, body mass index; CIT, cold ischemic time; EAD, early allograft dysfunction; GWR, graft weight ratio; MELD, model for end‐stage liver disease; NLR, neutrophil to lymphocyte ratio; PLR, platelet to lymphocyte ratio; RBC, red blood cell; WIT, warm ischemic time.

* Indicates that *p* < 0.05.

**TABLE 2:** Changes of major cell components in liver transplantation by blood routine.

	Non‐EAD (*n* = 74)		EAD (*n* = 35)		*p*‐value	
Cell type	Value (mean ± SD)	Ratio (/WBC)	Value (mean ± SD)	Ratio (/WBC)	Number	Ratio
Pre‐transplant						
RBC (*10^12/L)	3.15 ± 0.93	‐	3.25 ± 0.78	‐	0.60	‐
WBC (*10^9/L)	5.43 ± 5.32	‐	6.98 ± 5.78	‐	0.17	‐
Lymphocyte	0.84 ± 0.55	0.19 ± 0.11	0.79 ± 0.51	0.15 ± 0.086	0.70	0.045*
Monocyte	0.51 ± 0.56	0.093 ± 0.036	0.60 ± 0.53	0.093 ± 0.038	0.44	1.00
Neutrophil	3.93 ± 4.52	0.69 ± 0.13	5.51 ± 5.28	0.74 ± 0.11	0.11	0.048*
Platelet (*10^9/L)	67.27 ± 52.07	‐	71.69 ± 57.98	‐	0.69	‐
Post‐transplant						
RBC (*10^12/L)	2.64 ± 0.55	‐	2.62 ± 0.54	‐	0.84	‐
WBC (*10^9/L)	7.75 ± 5.06	‐	8.39 ± 4.21	‐	0.52	‐
Lymphocyte	0.39 ± 0.26	0.057 ± 0.036	0.40 ± 0.26	0.050 ± 0.026	0.85	0.28
Monocyte	0.44 ± 0.31	0.061 ± 0.025	0.50 ± 0.45	0.057 ± 0.029	0.47	0.56
Neutrophil	6.86 ± 4.64	0.87 ± 0.060	7.40 ± 3.72	0.88 ± 0.054	0.54	0.53
Platelet (*10^9/L)	57.82 ± 50.89	‐	47.66 ± 31.36	‐	0.28	‐

Abbreviations: EAD, early allograft dysfunction; SD, standard deviation; RBC, red blood cell; WBC, white blood cell.

* Indicates that *p* < 0.05.

**TABLE 3:** Dynamic peripheral immune cell profiling in liver transplantation.

	Non‐EAD (*n* = 74)		EAD (*n* = 35)		*p*‐Value	
Cell type	Absolute Number (*10^6/L) (Mean ± SD)	Ratio (/CD45+ cells)	Absolute Number (*10^6/L) (Mean ± SD)	Ratio (/CD45+ cells)	Number	Ratio
Pre‐transplant						
CD45+ cells	5323.35 ± 4547.74	‐	736.66 ± 88.38	‐	0.49	‐
CD3+ T cells	2914.39 ± 2355.98	0.71 ± 0.014	527.06 ± 74.56	0.15 ± 0.025	0.49	0.13
CD4+ T cells	306.61 ± 23.55	0.391 ± 0.014	262.91 ± 33.95	0.35 ± 0.014	0.29	0.054
CD8+ T cells	199.92 ± 24.18	0.24 ± 0.011	201.63 ± 33.58	0.24 ± 0.017	0.97	0.76
NK cells	82.85 ± 9.14	0.11 ± 0.009	76.74 ± 13.50	0.10 ± 0.009	0.71	0.58
B cells	126.20 ± 16.38	0.16 ± 0.013	124.66 ± 12.24	0.22 ± 0.025	0.95	0.034*
Post‐transplant						
CD45+ cells	781.97 ± 406.14	‐	382.10 ± 43.53	‐	0.50	‐
CD3+ T cells	461.97 ± 242.07	0.58 ± 0.018	229.80 ± 36.91	0.56 ± 0.030	0.51	0.46
CD4+ T cells	126.28 ± 10.67	0.34 ± 0.013	117.11 ± 16.60	0.30 ± 0.018	0.64	0.084
CD8+ T cells	81.85 ± 9.59	0.21 ± 0.011	84.34 ± 13.97	0.21 ± 0.018	0.88	0.98
NK cells	34.97 ± 5.83	0.092 ± 0.010	23.40 ± 3.31	0.065 ± 0.007	0.19	0.024*
B cells	115.74 ± 11.41	0.31 ± 0.020	125.86 ± 12.62	0.37 ± 0.031	0.59	0.10

Abbreviations: EAD, early allograft dysfunction; SD, standard deviation; NK cell, natural killer cell.

* Indicates that *p* < 0.05.

We analysed the correlation between the NK cell shift and the clinical characteristics associated with EAD. The NK cell shift was unrelated to donor BMI, graft weight, or fatty liver change (*p* > 0.05). However, it was linearly associated with CIT; prolonged CIT indicated a significant reduction of peripheral NK cell proportion (*p* = 0.016, Figure 1F). As prolonged CIT is a major cause of severe ischemic/reperfusion (I/R) injury in organ transplantation, this finding suggested that NK cell shift may be a critical immunological event related to I/R injury in the early phase after liver transplantation.

We monitored 16 patients with immune cell profiling data for at least 2 weeks after transplantation, and the trends of NK cell shift were depicted accordingly (Figure 1G). NK cell proportion declined 2–4 days after transplantation and gradually climbed. Of the 109 patients, nine did not recover from EAD on the seventh day after transplantation, and we defined it as 7d‐EAD. In other words, 7d‐EAD represents a subset of patients with higher severity and poorer outcomes. Interestingly, a relatively more significant NK cell shift was observed in these patients with 7d‐EAD (Figure 1H, *p* = 0.088). In addition, we also found that a significant NK cell shift (<2/3) was associated with relatively poorer survival after transplantation (*p* = 0.058, Figure 1I). Although not reaching statistical significance, this result may potentiate the use of the peripheral NK cell shift in the super‐early phase (2–4 days post‐transplant) to predict the recovery of EAD patients after transplantation.

### 2.2 NK cell was recruited into the liver after I/R treatment

**FIGURE 2:**
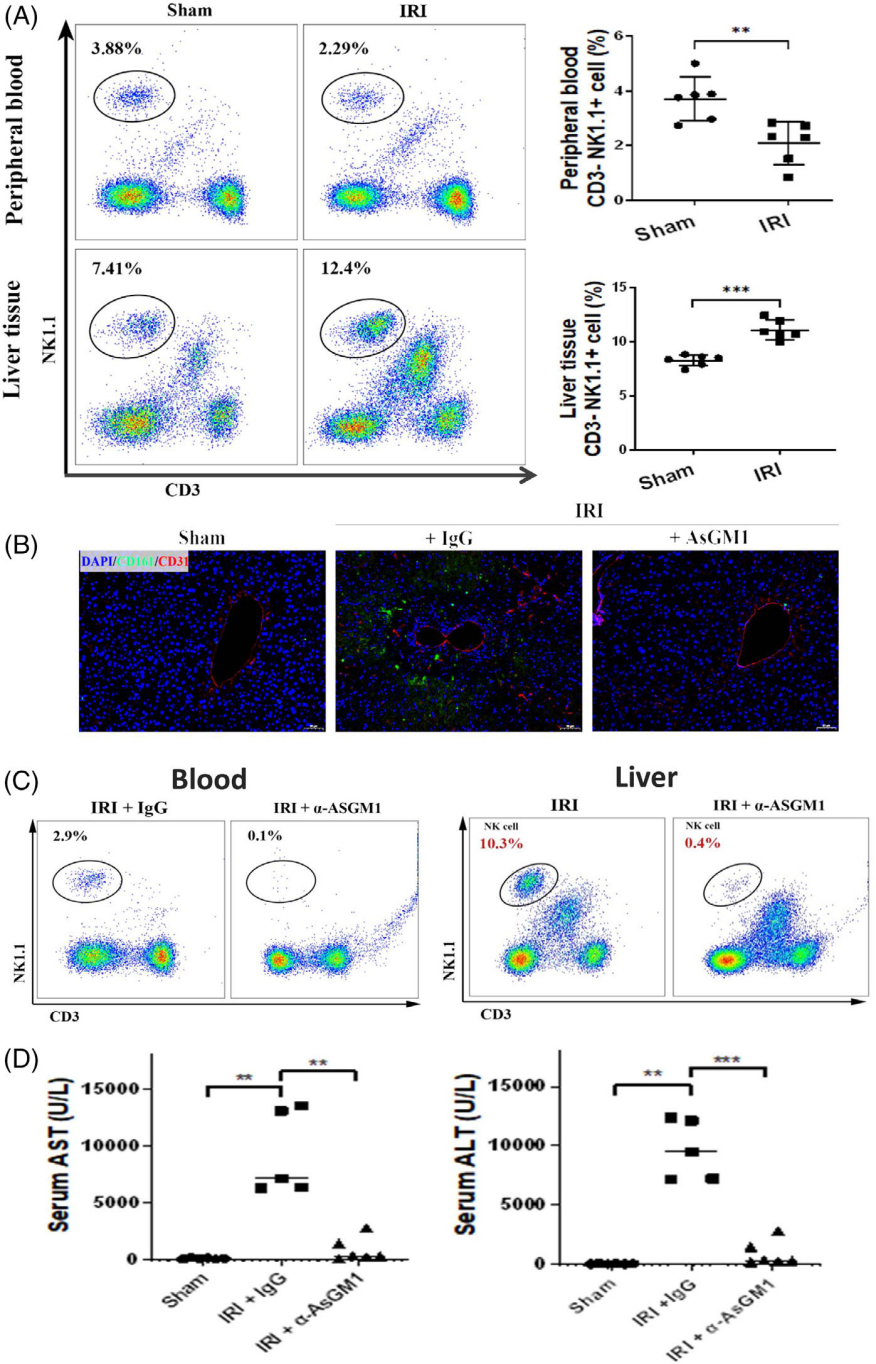
The role of NK cells in I/R injury. (A) After I/R treatment on the mouse liver, the proportion of NK cells in the peripheral blood decreased while the proportion of NK cells in the liver tissue increased significantly (*n* = 6). (B) Multiple immunofluorescences (DAPI + CD161 + CD31). NK cell infiltration increased after reperfusion, especially around the vessels. α‐AsGM1, an inhibitor for NK cells, decreased the infiltration of NK cells. (C) The circulating and hepatic NK cell proportions were significantly reduced using α‐AsGM1. (D) NK cell depletion by α‐AsGM1 can significantly reduce AST and ALT levels (*n* = 5 ~ 6). **p < 0.01; ***p < 0.001. ALT, alanine aminotransferase; AST, aspartate aminotransferase.

After the I/R treatment on the mouse liver models, the proportion of NK cells in the peripheral blood decreased. In contrast, the proportion of NK cells in the liver tissue increased significantly (*n* = 6, Figure 2A). Multiple immunofluorescences (DAPI + CD161 + CD31) showed that NK cell infiltration increased after reperfusion, especially around the vessels (Figure 2B). This indicated that in I/R injury, NK cells in the peripheral blood would be recruited into the liver tissue, consistent with our result that the peripheral NK cell shift occurred in EAD patients.

### 2.3 NK cell depletion alleviates hepatic I/R injury

**FIGURE 3:**
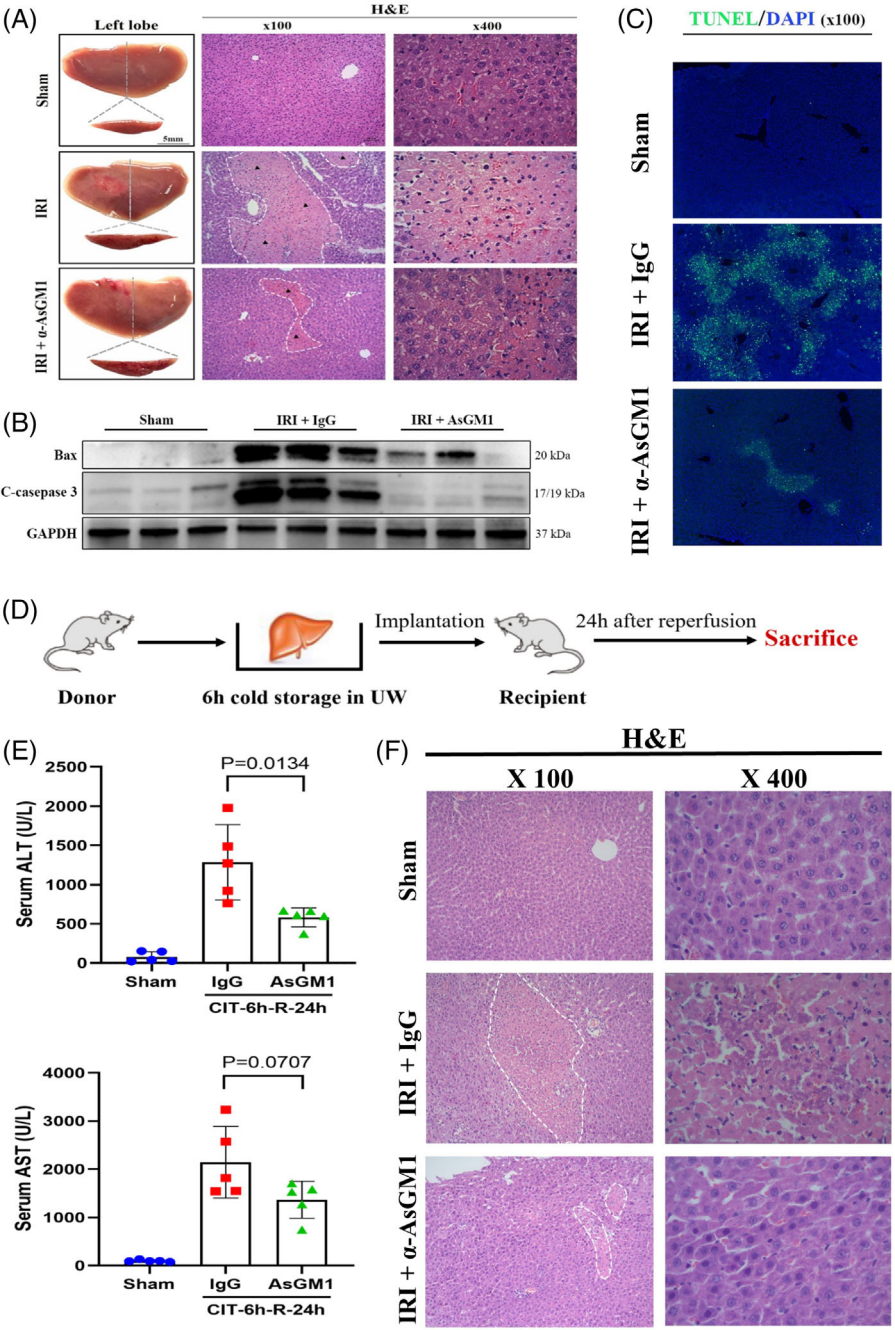
The impact of NK cell depletion on the liver after I/R treatment and liver transplantation. (A) Gross and microscopic images of the liver after I/R treatments. NK cell depletion group showed a significantly reduced area of necrosis by H&E staining. (B) Western‐blotting results for apoptotic proteins. NK cell depletion decreased hepatic BAX and cleaved‐caspase three levels in I/R injury. (C) TUNEL staining showed decreased hepatocyte apoptosis in I/R injury when NK cells were depleted. (D) Establishment of rat liver transplantation model with 6 h of cold preservation. (E) After transplantation, NK cell depletion significantly lowered the serum ALT and AST levels (*n* = 5). (F) Microscopic images of the transplanted liver graft tissue by H&E staining. A minor area of necrosis was observed in the NK cell depletion group by H&E staining. ALT, alanine aminotransferase; AST, aspartate aminotransferase.

The proportion of peripheral and hepatic NK cells was immensely reduced using the anti‐Asialo GM1 antibody (α‐AsGM1), an NK cell‐depleting antibody (Figure 2B,C). NK cell depletion alleviated I/R‐induced liver damage significantly, as evidenced by decreased AST and ALT levels (*n* = 5–6, Figure 2D), minor area of necrosis observed in haematoxylin–eosin (H&E) staining (Figure 3A), reduced apoptotic protein levels such as BAX and cleaved‐caspase 3 (Figure 3B), and decreased hepatocyte apoptosis in TdT‐mediated dUTP Nick‐End Labeling (TUNEL) staining (Figure 3C).

### 2.4 NK cell depletion improves liver function after transplantation

We then verified the effect of NK cell depletion in a rat liver transplantation model with prolonged CIT (6 h, *n* = 5, Figure 3D). NK cell depletion improved the liver function significantly after transplantation, with decreased serum ALT and AST levels (Figure 3E) and a minor area of necrosis seen in H&E staining (Figure 3F). This indicated that NK cell depletion could be a potential therapeutic strategy to protect liver grafts against I/R‐induced injury in liver transplantation.

### 2.5 Blockade of natural killer group 2 member D (NKG2D) inhibits I/R injury

**FIGURE 4:**
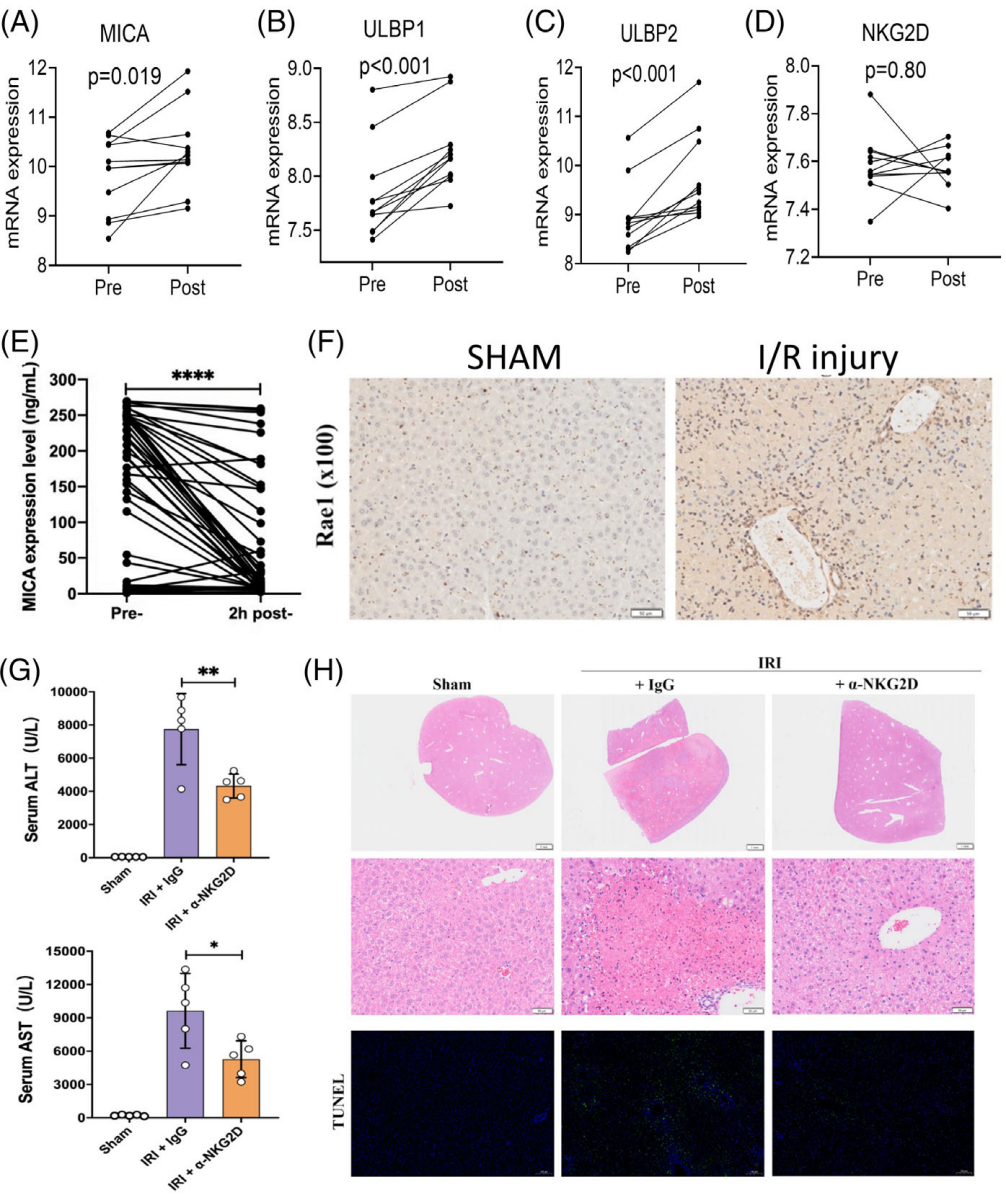
NKG2D mediated I/R injury. (A–D) Analysis of the GSE112713 dataset showed that the NKG2D ligands, including MICA, ULBP1 and ULBP2, were up‐regulated 60 min after reperfusion in liver transplantation. (E) Plasma MICA level was significantly decreased 2 h after reperfusion in liver transplantation. (F) Rae1, the MICA homologue in mice, was up‐regulated after I/R treatment. (G) NKG2D blockage decreased the ALT and AST level after I/R treatment. (H) H&E and TUNEL staining showed that NKG2D blockage could inhibit I/R injury. *p < 0.05; **p < 0.01; ***p < 0.001. ALT, alanine aminotransferase; AST, aspartate aminotransferase.

To further clarify the signalling pathway, we enrolled a gene expression dataset with 11 pairs of preserved liver graft tissue samples under pre‐ and post‐reperfusion during transplantation (GSE112713).^11^ The recruiting chemokines for NK cells were analysed. CXCL9, CXCL10 and CXCL11 were not significantly elevated after reperfusion in the liver graft. However, CX3CL1, a potent recruiter of NK cells, was significantly elevated (Figure S1). The role of NKG2D, a primary activating cell surface receptor in NK cells, was also studied. Although hepatic expression of NKG2D was comparable before and after reperfusion, its ligands, such as MICA, ULBP1 and ULBP2, were up‐regulated 60 min after reperfusion in liver transplantation (Figure 4A–D). We also collected peri‐operative plasma samples of liver transplantation to monitor the dynamic change of peripheral MICA. Interestingly, plasma MICA level was significantly decreased 2 h after reperfusion in liver transplantation (*p* < 0/001, Figure 4E). In vivo, Immunohistochemistry (IHC) showed that Rae1, the MICA homologue in mice, was up‐regulated in the liver after I/R treatment (Figure 4F). Most importantly, NKG2D blockage decreased the ALT and AST levels and inhibited liver tissue damage after I/R treatment (Figure 4G,H). The results indicate that the hepatic expression of NKG2D ligands and the subsequent activation of NKG2D may be a significant cause for NK cell shift in liver transplantation.

### 2.6 NK cell depletion alters a series of downstream immune cascades

**FIGURE 5:**
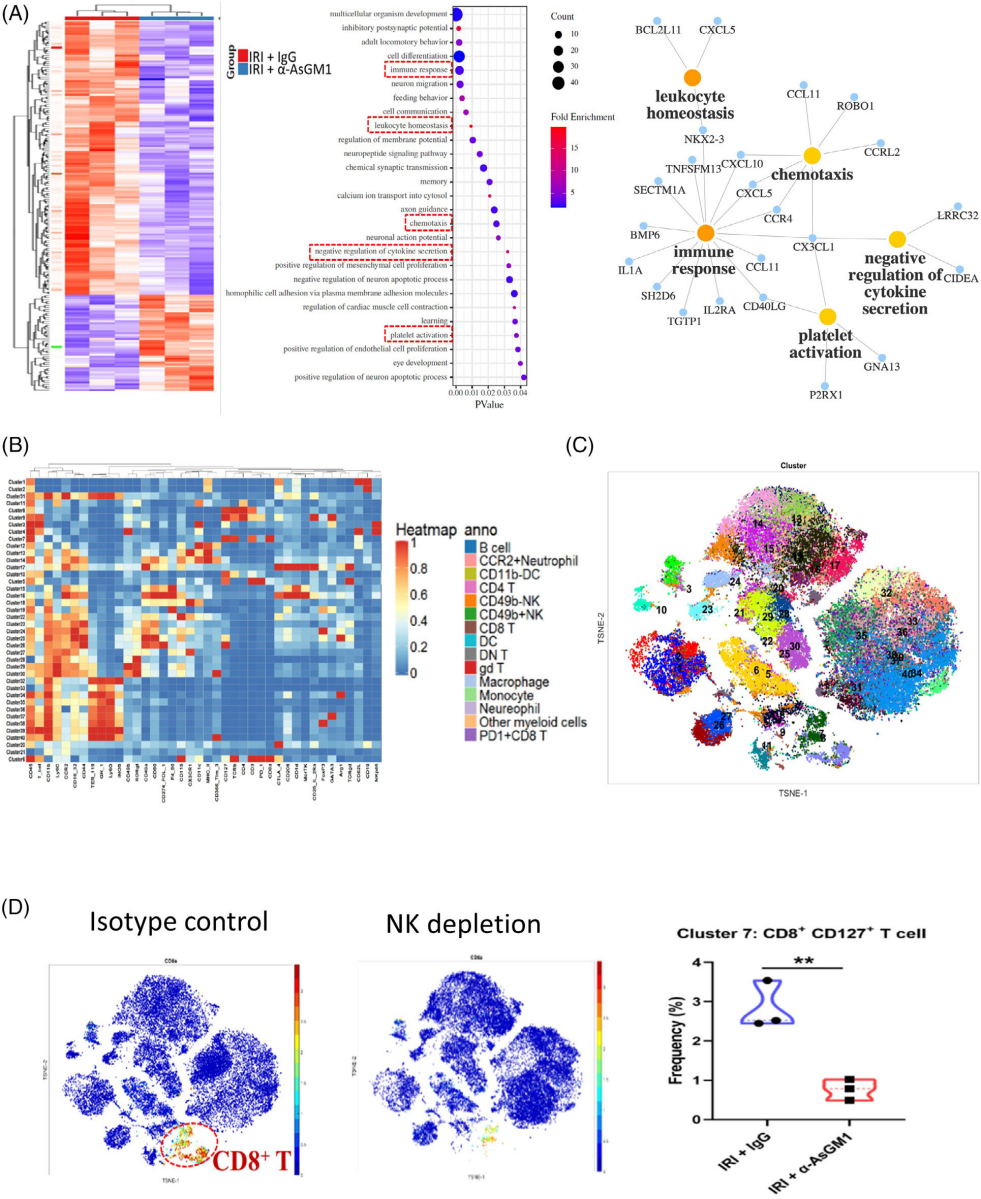
Signal transduction after NK cell depletion. (A) RNA‐sequencing comparing the control and NK depletion groups in I/R injury. Enrichment analysis revealed five immune‐related pathways after NK cell depletion: leukocyte homeostasis, chemotaxis, immune response, platelet activation and negative regulation of cytokine secretion. (B) CyTOF analysis of the liver tissue after I/R treatment. Forty clusters were identified. (C) A general viSNE graph of the infiltrating immune cells with the 40 clusters identified. (D) We compared the liver‐infiltrating immune cell components of the isotype control group (*n* = 3) and those in the NK cell depletion group (*n* = 3). CD8 + CD127+ T cell frequency was decreased in the NK depletion group. (E) The schematic diagram. **p < 0.01.

We further explored the downstream immune cascade triggered by NK cells in I/R injury by using RNA sequencing. When comparing mouse liver samples (after I/R treatments) of the isotype control group (*n* = 3) and those in the NK cell depletion group (*n* = 3), 465 differentially expressed genes were screened out (*p* < 0.05, fold change>1.5, Figure 5A). Pathway enrichment analysis showed that they were significantly related to immune‐related pathways such as negative regulation of cytokine secretion, immune response, leukocyte homeostasis, chemotaxis and platelet activation (*p* < 0.05). Analysis using the dataset (GSE112713) also showed an elevation of INF‐γ and IL‐10 after reperfusion, known as inflammatory cytokines secreted by NK cells (Figure S1).

We also compared the infiltrating immune cell components in the mouse liver (after I/R treatments) of the isotype control group (*n* = 3) and those in the NK cell depletion group (*n* = 3) by cytometry by time‐of‐flight (CyTOF). More than 4 million immune cells were screened, and 40 clusters were identified (Figure 5B). A general visualisation of *t*‐distributed stochastic neighbour embedding (viSNE) graph of the infiltrating immune cells is shown in Figure 5C. We also identified 94 pairs of solid correlations in between (Figure S2A). The viSNE graphs of the control group, NK cell depletion group and the merged diagram for contrast were shown in Figure S2B. The comparison identified three distinctive clusters between the two groups, namely, CD49b− NK1.1+ cells, CD49b + NK1.1+ cells and CD8+ CD127+ T cells (Figure 5D; Figure S1C). This demonstrated that NK cell depletion by α‐AsGM1 can inhibit both CD49b− and CD49b + NK cells and a unique subset of cytotoxic T cells, CD8+ CD127+ T cells.

## 3 DISCUSSION

The liver is enriched with innate immune cells, including NK cells, Kupffer cells, NKT cells, and so forth. Among them, NK cells are the most abundant lymphocyte population in the human liver.^12^ The relative abundance and unique characteristics of liver NK cells suggest they play essential roles in liver homeostasis.^13^ In a previous study based on the rat liver transplantation model, NK cells accounted for around 70% of the graft infiltrating lymphocytes 24 h after transplantation, and more than half were recipient‐derived.^14^ They also found that the proportion of NK cells in the peripheral blood decreases with a concurrent accumulation of NK cells in the graft. A similar phenomenon was observed in our study. A significant decrement of NK cells was detected in the early phase after liver transplantation, and then NK cell proportion gradually recovered to the pre‐transplant level. Interestingly, our study showed that the peripheral NK cell shift was more remarkable in EAD patients. This suggested that the NK cell shift might be a particular immunological response related to EAD in the acute phase after transplantation.

In our cohort, donor BMI, donor graft weight, liver fatty change and CIT were associated with the incidence of EAD after transplantation. These have been frequently reported as risk factors for EAD.^15^, ^16^, ^17^ Among them, only CIT was found to be related to the NK cell shift. Prolonged CIT is one of the critical elements in transplanted organ I/R injury.^18^ EAD was considered to be mediated mainly by I/R injury, as the presence of I/R injury confirmed by biopsy was closely associated with the incidence of EAD.^19^ It can activate systemic and hepatic inflammatory responses, such as innate macrophage activation.^20^ Our animal models of I/R injury demonstrated the recruitment of NK cells from peripheral blood into the reperfused liver. Besides that, the depletion of NK cells can alleviate I/R damage effectively and improve liver function after liver transplantation with prolonged CIT (6 h). The potential role of NK cells in I/R injury has already been mentioned in the literature.^21^ In a previous study by Kimura et al., donor liver resident NK cells played an essential role in I/R injury in allogeneic liver transplantation. Depleting donor liver, NK cells improved graft function.^22^ What is novel and exciting in our study is that we demonstrated the I/R injury‐related dramatic shift/recruitment of peripheral NK cells and depletion of recipient peripheral NK cells significantly improved liver function in rat liver transplantation. Also, this is the first study reporting the association between innate immune response and I/R‐induced severe complications, that is, EAD, in human liver transplantation. Inspired by the above‐mentioned findings, we explored how NK cells mediate liver graft damage.

Recently, damage‐associated molecular patterns (DAMPs) have been known to be involved in I/R injury.^23^ Under the stimulation of I/R stress, various patterns of recognition receptors can recognise the secreted ligands in DAMPs. Among them, NKG2D is an activating receptor that recognises stress molecules and is expressed steadily in most NK cells. Calabrese et al. revealed that NK cell NKG2D receptor ligation mediates pulmonary I/R injury.^24^ Here we identified elevated hepatic expression of NKG2D ligands after liver transplantation or I/R treatment and proved that NKG2D blockade could alleviate liver I/R injury. However, we also observed decreased FFPEMICA, a typical NKG2D ligand, in the plasma 2 h after reperfusion in liver transplantation, opposite to its hepatic expression pattern. A previous study demonstrated that the soluble MICA binds to NKG2D on NK cells and CD8 T cells and causes its internalisation followed by degradation, which is immunosuppressive.^25^ We suspect the soluble MICA is scavenged to avoid its immunosuppressive effect early after transplantation. After all, NK cell NKG2D ligations play a crucial role in I/R injury.

Moreover, the results of RNA sequencing showed that NK depletion could affect the expression of a series of genes related to an immune response, including IL1A and IL2RA. Interestingly, we also detected decreased hepatic CD8+ CD127 + T cells after I/R treatment by CyTOF. CD127 (also known as IL7RA), the receptor of IL7, is popularly regarded as the most critical cytokine for T‐cell homeostasis.^26^ Studies have proved that blockade of CD127 can decrease the number of CD4+ and CD8+ T cells and the associated cytokines, which in turn, halt the development of inflammatory diseases, including allergic asthma and arthritis.^26^, ^27^ Johnson and his colleagues found that tumour‐reactive T cells CD8+ T cells were activated dependent on cellular CD127 expression, indicating the potent activity and cytotoxicity of CD8+ CD127+ T cells.^28^ Therefore, it can be speculated that the NK− CD8+ CD127+ T cell signalling pathway may be an essential axis of graft injury after I/R treatments.

Here we drew the graphical abstract. NK cells were recruited and activated in the liver graft after I/R treatment, and this biological process may be partially mediated by the NKG2D ligation. The activated NK cells will then trigger an immune cascade and stimulate the CD8+ CD127+ T cell. It will finally induce potent hepatocytes apoptosis and result in EAD after liver transplantation.

However, there are limitations in this study. First, the sample size is relatively small. Various immunomodulatory treatments were conducted immediately after transplantation (such as basiliximab, steroid, tacrolimus and mycophenolate), which may also affect particular immune subsets and might be confusing. A larger cohort, as well as subgroup analysis, is needed for further validation. Second, Human NK cells are composed of two principal subsets, that is, CD56 (dim) CD16+ and CD56 (bright) CD16+/− subsets.^29^ They are reversely distributed in human blood and liver and may play distinctive roles in liver transplantation. Moreover, the NKT cells, which profoundly impact liver I/R injury,^30^ should not be ignored. However, the automatic gating of CD3/4/8/16/19/45/56 in our routine tests cannot identify these subsets. Moreover, we identified CD8+ CD127+ T cells as related to NK cells in I/R injury. More evidence shall be collected to ensure its impact on graft injury and its interactions with NK cells.

In conclusion, peripheral NK cell shift is associated with the incidence of EAD and may affect the outcome after liver transplantation. NK cell is a significant effector responsible for immune cascades in the early phase after reperfusion. Inhibition of NK cells or blockade of its surface‐activating receptor NKG2D effectively alleviates liver graft injury and can be a novel therapeutic strategy for EAD in liver transplantation.

## 4 SUBJECTS AND METHODS

### 4.1 Patients

This study enrolled patients undergoing donation after circulatory death liver transplantation in the First Affiliated Hospital, Zhejiang University School of Medicine, from April 2017 to October 2019. After excluding paediatric liver transplantation, combined transplantation, split liver transplantation, living donor liver transplant and those lacking essential data, 109 subjects were finally analysed. Recipients were monitored closely via the proper follow‐up system and the outpatient service from hospital discharge to the last follow‐up visit. The routine post‐transplant immune‐suppression protocol consisted of tacrolimus, basiliximab and mycophenolate mofetil. Basiliximab was administrated on post‐transplantation day 1 and day 4. Daily mycophenolate mofetil intake began from day 1 and tacrolimus from day 4 to maintain a stable liver graft function. 500 mg methylprednisolone was administrated during the operation. It was usually avoided after transplantation unless for cases such as ABO‐incompatible transplantation, transplantation for autoimmune liver diseases, graft cholestasis, severe impairment of the immune system and so forth.

The research was carried out according to The Code of Ethics of the World Medical Association (Declaration of Helsinki). The clinical part of study was registered in the Research Registry (https://www.researchregistry.com/, identifying number: researchregistry9530). Signed informed consent was obtained from all the patients. The study protocol was also approved by the Human Ethics Committee of the hospital (#2020‐051).

### 4.2 Mouse I/R model

Male mice (C57BL/6J) were obtained from the Vital River Laboratories (Beijing, China), with each weighing around 20 g. The mice fasted overnight before I/R treatments. According to the study design, the pre‐treatments included^1^ Isotype control: peritoneal injection of 50 μL InVivoMAb polyclonal rabbit IgG 3 days before I/R treatment^2^; NK cell depletion: peritoneal injection of 50 μL α‐AsGM1 3 days before I/R treatment^3^; NKG2D blockade: peritoneal injection of 200 μg InVivoMAb anti‐mouse NKG2D (CAT# 780320 J2) 7 days and 1 day before I/R treatment, respectively. I/R treatment in mouse liver was performed following Pringle's manoeuvre by clamping the portal triad (hepatic artery, portal vein and biliary tract) for 90 min and removing the clamp. The mice were sacrificed 6 h after I/R treatment, and their serum and liver tissue were obtained for detection.

### 4.3 Rat liver transplantation model

Male SD rats were obtained from the Vital River Laboratories (Beijing, China), weighing around 250 g. The rats fasted overnight before liver transplantation. The experimental rats were divided into three groups^1^: Sham treatment group (*n* = 5)^2^; Isotype Group: tail vein injection of 100 μL InVivoMAb polyclonal rabbit IgG in the recipient rats 3 days before transplantation (*n* = 5)^3^; NK cell depletion group: tail vein injection of 100 μL α‐AsGM1 in the recipient rats 3 days before transplantation (*n* = 5). Rat orthotopic liver transplantations were performed as described previously in our centre.^31^ All liver grafts underwent 6 h of cold storage in the University of Wisconsin solution for sufficient I/R injury after revascularisation. The rats were sacrificed 24 h after transplantation, and their serum and liver tissue were obtained for detection.

### 4.4 Serum transaminase assay

Mouse and rat serum ALT and AST levels were detected using a Fully Automatic Biochemical Analyzer (BS‐220, Mindray).

### 4.5 Flow cytometry analysis

Peripheral immune cell profiling (CD3/4/8/16/19/45/56) was performed using a BD MultiTEST IMK kit according to the instruction manual. Gating in flow cytometry was performed by BDFACSCanto™ automatically. The results will be verified and fine‐tuned by a professional technician. The tests were routinely performed on the first day of admission to the transplant centre transplantation. All of them should be within 30 days before the transplant. The patient's condition should maintain stable without major interventions, such as an artificial liver support system during the interval between pre‐transplant tests and the day of liver transplantation. Otherwise, the case should be excluded. The tests would then be scheduled in the intensive care unit twice a week after transplantation (3–4 days' intervals).

Mouse peripheral blood mononuclear cells and liver tissue infiltrating lymphocytes were isolated separately and incubated with 7‐AAD (420404, Biolegend, CA), fluorescein 488‐conjugated anti‐CD3 antibody and phycoerythrin (PE)‐conjugated anti‐NK1.1 antibody. The incubated cells were washed three times with phosphate‐buffered saline. 7‐AAD was used to exclude the dead cells before analysis. CD3 and NK1.1 level was determined based on the 1 × 10^5^ cellular events using a 4‐colour FC500 flow cytometer (Beckman‐Coulter, Miami, FL, USA).

### 4.6 H&E and IHC staining

Tissue specimens from mouse and rat liver grafts were fixed in 4% formalin, embedded in paraffin and stored at 4°C. For further examination, the samples were sliced into 5 μm‐thick sections. The slides were stained with H&E. IHC using MICA antibody(Cat# BS8377, Bioworld Technology) was performed as previously described.^32^

### 4.7 Multiplex immunofluorescence staining

Multiplex staining of formalin‐fixed paraffin‐embedded tissue was performed after deparaffinised and rehydrating, antigen retrieval, spontaneous fluorescence quenching and BSA blocking. CD161 (Cat# 39197S; CST) and CD31 (Cat# AF3628; R&D SYSTEMS) antibodies were sequentially applied, followed by species‐corresponded secondary antibody incubation. Nuclei were stained with DAPI after all the antigens had been labelled. The stained slides were scanned by the confocal microscope (Olympus).

### 4.8 TUNEL detection

Hepatocyte apoptosis in the 5 μm tissue specimen was detected using In Situ Cell Death Detection Kit (11684817, Roche) according to its manual.

### 4.9 Western Blot

Total proteins were extracted from liver tissues. For the detection of apoptotic proteins, the anti‐Bax Antibody (1:1000), anti‐Cleaved Caspase‐3 Antibody (1:1000) and anti‐GAPDH Antibody (1:1000) were used. Immunodetection was performed as previously described.^32^

### 4.10 Elisa test

Plasma MICA concentration was tested using the MICA Elisa kit (OSD‐H2405, Ousaid Biotechnology Co., Ltd) according to its manual.

### 4.11 RNA sequencing

After the extraction of total RNA was completed, mRNA was isolated by Oligo Magnetic Beads. Libraries were generated using the NEBNext Ultra™ RNA Library Prep Kit (BGI‐Tech, Shenzhen, China) for the Illumina system following the manufacturer's instructions. Sequencing was conducted using the Illumina Hiseq XTEN platform.

### 4.12 CyTOF

CyTOF analysis was performed by PLTTech Inc. (Hangzhou, China) according to the protocol described previously.^33^ In brief, mouse liver tissue was dissociated into single cells with DNAase, collagenase IV, and hyaluronidase (Sigma‐Aldrich, Saint Louis, MO, USA). Immune cells were concentrated using Percoll density gradient media (Sigma‐Aldrich), while red blood cells were removed using ACK Lysing Buffer (Sigma‐Aldrich). Qualified samples were gathered in blocks and stained for 30 min with a panel of 42 antibodies, followed by overnight fixation. Permeabilisation buffer was applied, and the cells were incubated in an intracellular antibody mixture. The cells were rinsed, and the signals were detected using a CyTOF system (Helios, Fluidigm, South San Francisco, CA, USA). The types of immune cells were identified via nonlinear dimensionality reduction (viSNE) followed by density‐based clustering.

### 4.13 Data analysis

The results were analysed and graphed by R Version 3.6.1 (http://cran.r-project.org), including program package *vioplot, correlation* and *pheatmap*, SPSS (Version 22.0, Inc., IL, USA) and Prism 6 (GraphPad Software, CA). Kaplan–Meier method was used for survival analysis. Multivariate binary logistic regression was employed to identify independent risk factors related to EAD (backward, Wald). Student's *t*‐test or one‐way ANOVA test was used, when available, for comparisons between groups. A *p*‐value below 0.05 was considered statistically significant.

## AUTHOR CONTRIBUTIONS

*Conception and design*: Xiao Xu, Di Lu and Shusen Zheng. *Acquisition of data*: Xinyu Yang, Linhui Pan, Zhengxing Lian and Modan Yang. *Analysis and interpretation of data*: Di Lu, Linhui Pan, Jianyong Zhuo and Zuyuan Lin. *Drafting and revising the article*: Linhui Pan, Winyen Tan, Qiang Wei and Jun Chen. Final approval of the version to be published: Xiao Xu.

## FUNDING INFORMATION

This work was supported by the Key Program, National Natural Science Foundation of China (No. 81930016), National Key Research and Development Program of China (No. 2021YFA1100500), Young Program of National Natural Science Funds of China (No. 82000617) and Key R&D Program of Zhejiang (No. 2022C03108).

## CONFLICT OF INTEREST STATEMENT

All authors declare no conflict of interest.

## Supporting information


**FIGURE S1.** Changes in the recruiting chemokines of NK cells and the secreted cytokines after hepatic reperfusion.

**FIGURE S2.** CyTOF analysis for the liver tissue after I/R treatment. (A) Correlation analysis map of all clusters. (B) The viSNE graphs of the isotype control group, the NK cell depletion group and the merged viSNE graph for contrast. (C) We compared the liver‐infiltrating immune cell components of the isotype control group (*n* = 3) and those in the NK cell depletion group (*n* = 3). The frequency of CD49b− NK1.1+ NK cells and CD49b + NK1.1+ NK cells were reduced by NK cell depletion.

## Data Availability

The data generated and analysed during the current study are available from the corresponding author upon reasonable request.
