# Supplementary figures and images for: Dynamic immune cell profiling identified natural killer cell shift as the key event in early allograft dysfunction after liver transplantation
Source: Cell Prolif. 2023 Oct 31;57(4):e13568. doi: 10.1111/cpr.13568 (PMC10984105; doi:10.1111/cpr.13568)

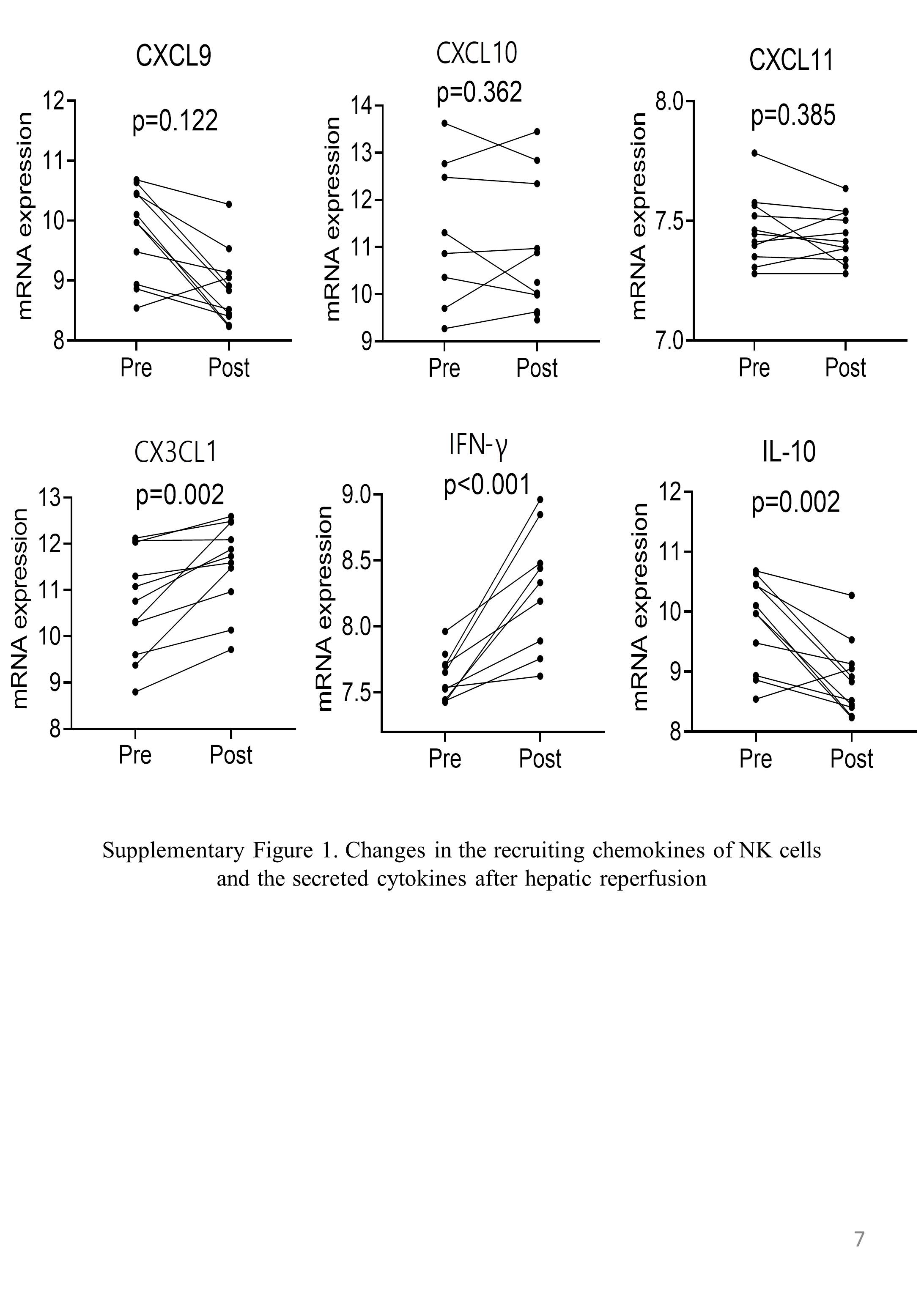

Supplement: Supplementary file 1 — FIGURE S1. Changes in the recruiting chemokines of NK cells and the secreted cytokines after hepatic reperfusion. [file CPR-57-e13568-s001.jpg]

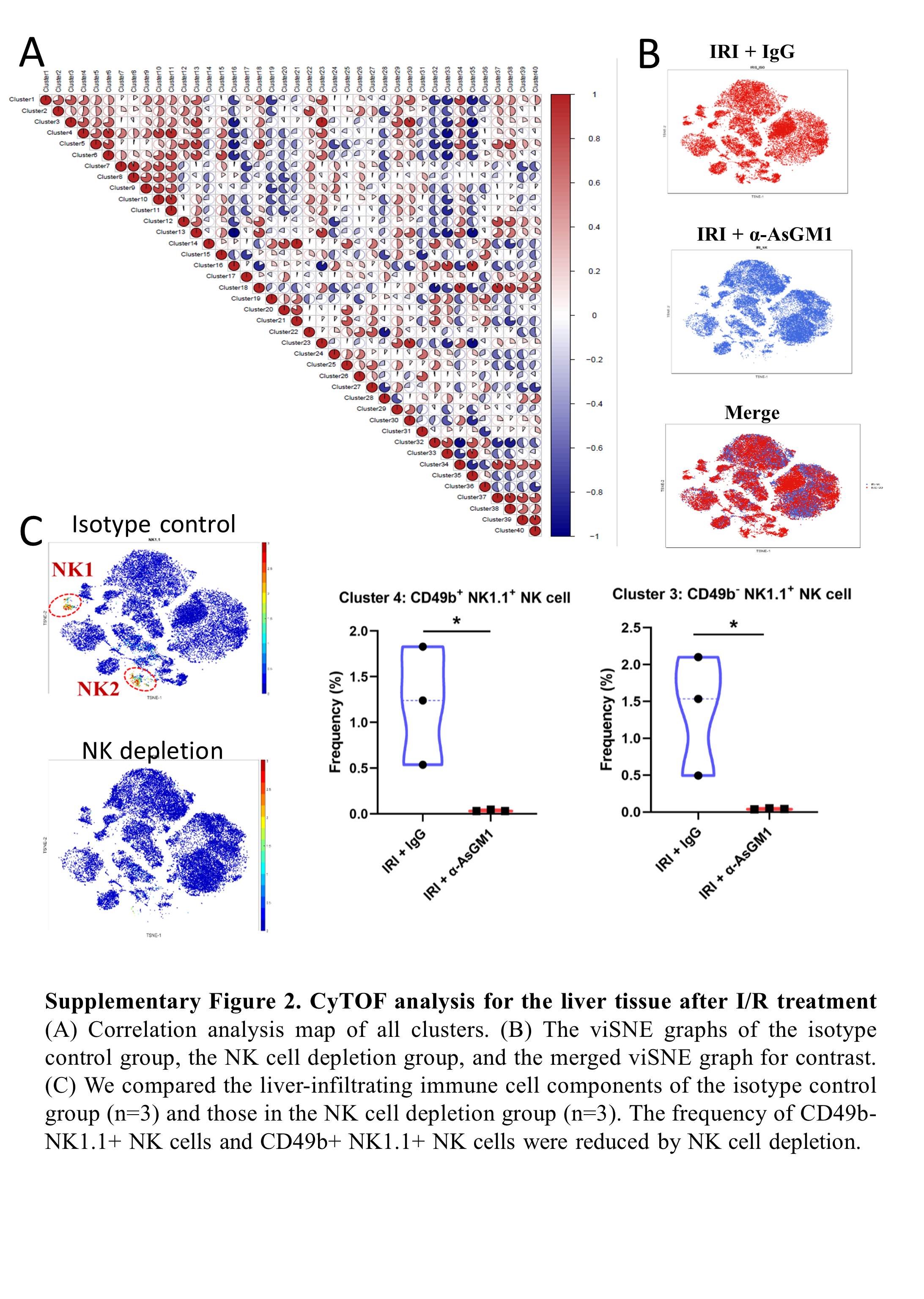

Supplement: Supplementary file 2 — FIGURE S2. CyTOF analysis for the liver tissue after I/R treatment. (A) Correlation analysis map of all clusters. (B) The viSNE graphs of the isotype control group, the NK cell depletion group and the merged viSNE graph for contrast. (C) We compared the liver‐infiltrating immune cell components of the isotype control group (n = 3) and those in the NK cell depletion group (n = 3). The frequency of CD49b− NK1.1+ NK cells and CD49b + NK1.1+ NK cells were reduced by NK cell depletion. [file CPR-57-e13568-s002.jpg]
